# Automated microscopy for routine malaria diagnosis: a field comparison on Giemsa-stained blood films in Peru

**DOI:** 10.1186/s12936-018-2493-0

**Published:** 2018-09-25

**Authors:** Katherine Torres, Christine M. Bachman, Charles B. Delahunt, Jhonatan Alarcon Baldeon, Freddy Alava, Dionicia Gamboa Vilela, Stephane Proux, Courosh Mehanian, Shawn K. McGuire, Clay M. Thompson, Travis Ostbye, Liming Hu, Mayoore S. Jaiswal, Victoria M. Hunt, David Bell

**Affiliations:** 1 Universidad Peruana Cayetano Heredia, Laboratorio de Malaria, Laboratiorios de Investigacion y Dessarrollo, Facultad de Ciencias y Filosofia, Av. Honorio Delgado 430 SMP, Lima, Peru; 2 Intellectual Ventures, 3150 139 AVE SE, Bellevue, WA 98005 USA; 3 Shoklo Malaria Research Unit, 68/30 Bantung Road, Mae Sot, PO Box 46, Tak, 63110 Thailand

**Keywords:** Malaria, Convolutional neural networks, Microscopy, Digital microscopy, Artificial intelligence

## Abstract

**Background:**

Microscopic examination of Giemsa-stained blood films remains a major form of diagnosis in malaria case management, and is a reference standard for research. However, as with other visualization-based diagnoses, accuracy depends on individual technician performance, making standardization difficult and reliability poor. Automated image recognition based on machine-learning, utilizing convolutional neural networks, offers potential to overcome these drawbacks. A prototype digital microscope device employing an algorithm based on machine-learning, the Autoscope, was assessed for its potential in malaria microscopy. Autoscope was tested in the Iquitos region of Peru in 2016 at two peripheral health facilities, with routine microscopy and PCR as reference standards. The main outcome measures include sensitivity and specificity of diagnosis of malaria from Giemsa-stained blood films, using PCR as reference.

**Methods:**

A cross-sectional, observational trial was conducted at two peripheral primary health facilities in Peru. 700 participants were enrolled with the criteria: (1) age between 5 and 75 years, (2) history of fever in the last 3 days or elevated temperature on admission, (3) informed consent. The main outcome measures included sensitivity and specificity of diagnosis of malaria from Giemsa-stained blood films, using PCR as reference.

**Results:**

At the San Juan clinic, sensitivity of Autoscope for diagnosing malaria was 72% (95% CI 64–80%), and specificity was 85% (95% CI 79–90%). Microscopy performance was similar to Autoscope, with sensitivity 68% (95% CI 59–76%) and specificity 100% (95% CI 98–100%). At San Juan, 85% of prepared slides had a minimum of 600 WBCs imaged, thus meeting Autoscope’s design assumptions. At the second clinic, Santa Clara, the sensitivity of Autoscope was 52% (95% CI 44–60%) and specificity was 70% (95% CI 64–76%). Microscopy performance at Santa Clara was 42% (95% CI 34–51) and specificity was 97% (95% CI 94–99). Only 39% of slides from Santa Clara met Autoscope’s design assumptions regarding WBCs imaged.

**Conclusions:**

Autoscope’s diagnostic performance was on par with routine microscopy when slides had adequate blood volume to meet its design assumptions, as represented by results from the San Juan clinic. Autoscope’s diagnostic performance was poorer than routine microscopy on slides from the Santa Clara clinic, which generated slides with lower blood volumes. Results of the study reflect both the potential for artificial intelligence to perform tasks currently conducted by highly-trained experts, and the challenges of replicating the adaptiveness of human thought processes.

**Electronic supplementary material:**

The online version of this article (10.1186/s12936-018-2493-0) contains supplementary material, which is available to authorized users.

## Background

Timely and accurate diagnosis of malaria is critical for effective treatment and to prevent transmission in communities. Microscopic examination remains the gold standard for laboratory confirmation of malaria. Specifically, light microscopy of Romanowski-stained blood films introduced by Laveran over 130 years ago [1, 2] remains critical in malaria case management and as a reference standard for research and disease monitoring. However, light microscopy remains a source of uncertainty because it is highly dependent on individual technician performance [3]. Antigen-detecting rapid diagnostic tests (RDTs) have become the predominant diagnostic tool for case management since their introduction over 20 years ago [4, 5], but they too have limitations in specificity and sensitivity, in species differentiation, and in their inability to provide accurate quantitation [6]. Molecular methods are improving, becoming cheaper and simpler [7], but remain confined to specialized laboratories.

Visualization of parasites is not subject to issues of antigen persistence, or to genetic mutations that limit the applicability of the major histidine-rich protein 2 (HRP2)-detecting antigen-based assay for *Plasmodium falciparum*, for example in Peru [8]. However, microscopy has major shortfalls in identifying, quantifying, and differentiating parasites from artifacts, and differentiating species. Furthermore, microscopy requires training, patience, competence, and well-managed workflows [9]. Even with skilled technicians, there is variability, which presents challenges in making reliable comparisons over time and geography [10, 11]. Low accuracy in case management leads to direct costs in morbidity and potential death, while inaccuracy in research leads to misleading results and resultant poor decision-making in product evaluations, resource allocations, and planning. While considerable efforts have been made in recent years to address quality assurance inadequacies [10], such programmes are expensive and limited in scope; others, such as the essential activities in the World Health Organization (WHO) therapeutic efficacy survey (TES) monitoring programme [12], rely on highly accurate film interpretation, and thus are difficult to scale.

The application of digital image recognition to malaria microscopy, using artificial intelligence algorithms to replace or supplement the human factor in blood film interpretation, have been attempted, usually on thin films [13, 14]. Some have coupled this with automated non-Romanowski staining techniques aimed at reducing variability in film preparation [15–17] in efforts to address the barriers in training, facility, and quality reagent requirements of manual Giemsa staining [18].

Standardization of blood films can simplify development and operation of image-recognition algorithms. However, automated staining with Romanowski stains cannot be achieved with simple equipment. Fluorescent staining, although it enables automation, adds new challenges in species differentiation, and also adds costs in new reagents, devices and supply lines. Devices based on non-Romanowski staining will be unable to read the 200 million plus slides currently produced each year for malaria case management [19], and will, therefore, require major changes in procedures closer to patient care in order to reach scaled implementation. As an alternative to the methods described above, recent developments in machine-learning techniques, based on the use of convolutional neural networks (a form of artificial intelligence in which an algorithm automatically extracts useful visual features to analyse images), open new possibilities for automated recognition of malaria parasites in standard Giemsa-stained blood films and for coping with inherent variability in film preparation.

The core of the Autoscope algorithm is based on convolutional neural networks (CNN)—a new and rapidly evolving field. Convolutional neural networks have been applied to a number of image detection and classification tasks on which they have achieved human-level performance [20–25]. CNN algorithms ‘learn’ based on analysing large numbers of objects classified by humans into different categories, and make ‘decisions’ on which features or patterns best distinguish these object categories. A full description of the Autoscope image analysis architecture is provide in the supplemental information section. Algorithm performance depends on the accuracy of the original object classification by trained microscopists, the number of training objects, and the similarity of training images to test images.

This paper reports diagnostic accuracy of Autoscope, a prototype parasite recognition system for images of Giemsa-stained malaria blood films, based on deep neural networks and other machine learning methods [26]. The Autoscope incorporates automated scanning of Giemsa-stained blood films and parasite-detection software to identify and count malaria parasites, and uses an algorithm trained on a broad range of blood films from geographically-diverse sites. The Autoscope scans with a digital camera based on a standard 100× oil-immersion objective optical train. Details of the automated scanning microscope hardware are given in the Additional files section, and have been described previously [26]. Annotation methods are described in the Additional file 1. The algorithm was trained on two species, *P. falciparum* and *Plasmodium vivax,* confined to thick film interpretation. The algorithm is intended to form the foundation for a more broadly applicable system to consistently mimic the competency of an expert microscopist and standardize film interpretation between sites and across time.

**Fig. 1:**
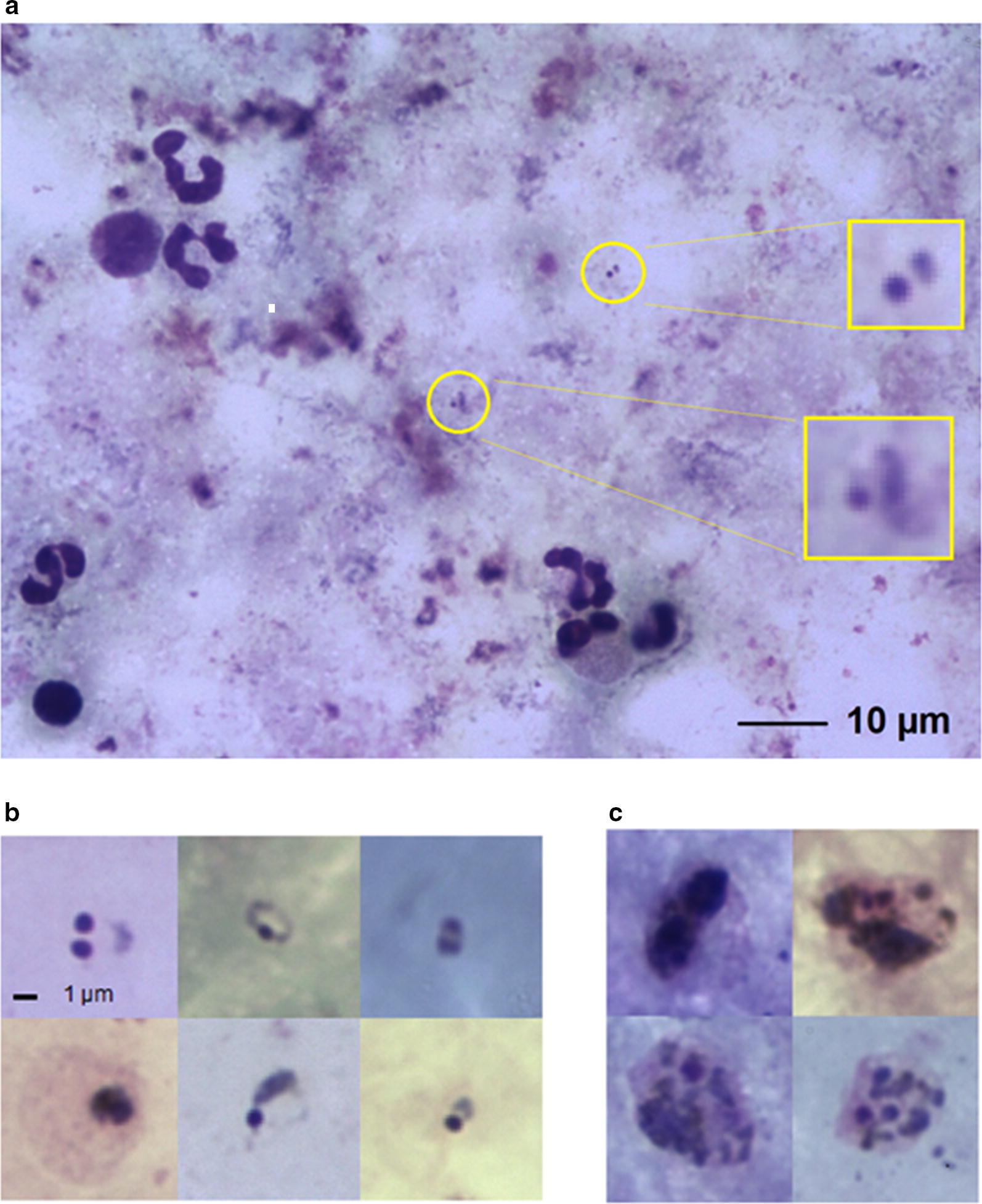
Typical thick film microscope images. **a** A field-of-view image containing only two parasites, indicated by yellow circles with enlargements. **b** Malaria parasites ring forms. **c** Malaria parasite late stages

The Autoscope algorithm had previously undergone one round of field optimization at Shoklo Malaria Research Unit (SMRU) in Thailand, which also conducted the external quality control (EQC) for this study. The device scans approximately 2.5 mm^2^ of Giemsa-stained thick blood film which corresponds to approximately 0.1 µL of blood on a thick blood film conforming to the WHO standard [10]. The algorithm calibration assumes ≥ 0.1 µL of blood imaged and the device issues a warning of unreliable results if it counts a low number of white blood cells (WBCs). When imaging thick blood films, Autoscope images 9 vertically-stacked slices for each of 324 Fields-of-View (FoV). A portion of a typical thick film is shown in Fig. 1a. Thumb nails of malaria parasites are shown in Fig. 1b, c. The Autoscope algorithm then processes the captured images to detect both ring and late-stage malaria parasites and returns a diagnosis and parasite quantitation. Although Autoscope can image thick or thin films, the algorithm version used in this field study only operated on thick films, distinguishing species only as *P. falciparum* or *P. vivax*.

This paper describes a study to determine the equivalency of the diagnostic performance of the prototype Autoscope compared to a microscopist in a field setting, relative to polymerase chain reaction (PCR) as reference.

## Methods

This prospective study was conducted in the district of San Juan Bautista in the province of Maynas, department of Loreto in Peru. San Juan, with population 145,238 [27], is an area of high risk of malaria transmission in Loreto and consists of three zones: urban, peri-urban, and rural populations, the latter two of which are high risk areas for malaria transmission due to the presence of the primary malaria vector *Anopheles darlingi*.

Two health care sites with diagnostic services enrolled patients for the study: San Juan de Miraflores Health Centre (San Juan), and Santa Clara de Nanay Health Post (Santa Clara). San Juan is a larger facility serving patients from various communities, including more distant, malaria-endemic communities, whereas Santa Clara provides more basic health care to a smaller, more remote population.

**Fig. 2:**
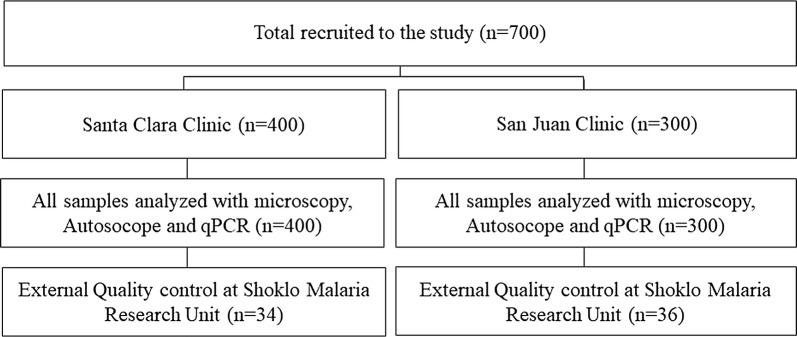
Flow chart of samples from study participants

A total of 700 individuals (400 from Santa Clara and 300 from San Juan) were recruited using consecutive sampling from April 2016 to July 2016 with the following inclusion criteria: (1) age between 5 and 75 years; (2) history of fever within the last 3 days, or elevated temperature (≥ 37.5 °C) on the day of admission; (3) informed consent. A finger prick blood sample was taken to create blood films for microscopy diagnosis, and additional drops of blood were spotted onto filter paper for subsequent qPCR analysis. After sampling, any patients with a malaria diagnosis using manual microscopy received treatment, administered by the Regional Malaria Control programme in accordance with Ministry of Health guidelines [28]. All samples were analysed with microscopy and Autoscope. A subset of 10% of samples were sent to SMRU for EQC (Fig. 2).

### Blood film reading by microscopy and Autoscope

Blood slides, each with both thin and thick films were stained with 10% Giemsa in phosphate buffer (pH 7.2) for 10 min, and air-dried as described in the national standard protocols [28]. At each clinic, three slides were prepared from each patient. Two of these slides were set aside for the participating health centre and for internal quality control. The third slide was used in the study and examined by both the microscopists and the Autoscope device.

At the clinics, detection of the malaria parasite, quantitation, and differentiation of the species were first performed by microscopy by clinic technicians (using both thick and thin films, according to protocols), and then by an Autoscope (using the same blood film and thick film only). Clinic technicians were blinded to Autoscope results. The slides were then sent to the Universidad Peruana Cayetano Heredia (UPCH) laboratory based in Iquitos (‘Morona’) for a quality-control reading by expert microscopists and a reading by a different Autoscope. The expert microscopists at Morona were blind to initial microscopy results. Expert microscopists were certified by the National Institute of Health from Peru.

Malaria diagnosis by Autoscope involves imaging the thick blood film, then running the diagnostic algorithm on the collected images. For imaging, the Giemsa-stained slide is inserted into the device, in the same way as a slide would be inserted into a 100× oil-immersion manual microscope. Diagnosis, species identification, quantitation, and mosaics of thumbnails of suspected parasite objects are automatically generated and outputted in an html report. Diagnosis thresholds on Autoscope are pre-specified, using results on a set of validation samples, with the goal of achieving > 90% specificity.

### Real-time PCR

Real-time qPCR was performed using a CFX Connect Real Time System (BioRad). Approximately 80 μL of blood was applied to a filter paper 3 M (Watman) and allowed to dry completely. Parasite DNA from a double 10 mm hole punch was extracted using the E.Z.N.A^®^ Blood DNA Mini Kit (Omega Bio-tek) according to the manufacturer’s instructions, except that Buffer ATL (Qiagen) and Proteinase K at 20 mg/mL (Qiagen) were used in the lysis step. Amplification was carried out in a 25 µL reaction volume containing 5 µL DNA, 12.5 µL of SYBR^®^ Green PERFECTA^®^ Fastmix (Quanta Biosciences) and 0.3 µM of each primer. A pair of primers was used to amplify the 18S rRNA gene sequences: PL1473F18 [5′-TAA CGA ACG AGA TCT TAA-3′] and PL1679R18 [5′-GTT CCT CTA AGA AGC TTT-3′]) [29]. The conditions for the qPCR consisted of an initial denaturation at 95 °C for 2 min followed by 45 cycles of amplification at 95 °C for 20 s, 52 °C for 20 s and 68 °C for 30 s and a final extension at 68 °C for 3 min, with fluorescence acquisition at the end of each extension step. After PCR amplification, a melting curve was performed to check the specificity of the amplicon, this consisted of a cycle at 95 °C for 10 s, followed by 68 °C for 2 min, and a gradual temperature increase of 0.5 °C/s up to 90 °C, with fluorescence measurement at each temperature transition. Each run included 2 positive controls (DNA sample from *P. vivax* and *P. falciparum*), a negative control (DNA from uninfected human), and a blank (nuclease-free water).

For all samples, the baseline and threshold were assigned for each run by the software BioRad CFX Manager 3.1. Samples were considered negative if the cycle threshold (Ct) value was greater than 39 or if the melt curve did not exceed the threshold or did not align with the positive control. Samples were considered positive when amplification curves crossed the threshold before cycle 39 and had the expected melt curve profile for each species (*P. falciparum* 73.5 °C ± 0.5 °C; *P. vivax* 77.5 °C ± 0.5 °C). Technicians performing qPCR were blinded to all results obtained from clinic sites.

### Quality control

SMRU received 70 paired field slides representing 10% of the Autoscope evaluation. Slides came from both San Juan and Santa Clara clinics. SMRU has been an early evaluator of the Autoscope and has expert microscopy capacity to diagnose multiple species.

### Analysis

Autoscope performance measures that were assessed included sensitivity, specificity, positive predictive value (PPV), negative predictive value (NPV), quantitation accuracy, and species ID accuracy. Performance was evaluated in two ways: (1) Diagnosis was compared to expert microscopy using PCR as reference; (2) Quantitation was directly compared to expert microscopy. Autoscope performance was parsed by WBC count because the detection algorithm assumes that 0.1 µL of blood has been imaged. Indeterminate tests were handled using the Obare Method [30], which is an excel-based tool to tabulate concordance of samples, and a technical data quality coordinator guaranteed completeness of the data.

For Autoscope performance, the Autoscopes at the two clinics of origin (San Juan, Santa Clara) were used. For expert microscopy performance, the microscopy readings from Morona were used. Microscopists at the clinics of origin produced very similar results; agreement between clinic microscopy diagnosis (including species) vs. Morona microscopy diagnosis was 97% (95% CI 95–98%) for the Santa Clara clinic, with 387 of 400 sample diagnoses in agreement. Agreement between clinic microscopy diagnosis *versus* Morona diagnosis was 99% (95% CI 98–100%) for the San Juan clinic, with 298 of 300 sample diagnoses in agreement.

Results from the two clinics are presented separately, to demonstrate variability of diagnostic accuracy per clinic. Point estimates and exact binomial confidence intervals for sensitivity, specificity, accuracy, PPV and NPV were calculated using the EpiR Package [31] in the R statistical environment [32].

The sample size of 700 was calculated as the number of samples required to characterize diagnostic performance (sensitivity and specificity) of the Autoscope with 5% margin of error, assuming 95% sensitivity and specificity, and 25% prevalence (one-sided exact binomial test, significance level α = 0.05, power 1 − β = 0.80).

## Results

### Peru field data

Autoscope performance differed between the clinics of origin of the slides processed, and by volume of blood imaged. San Juan thick films generally had greater volume of blood per unit area than Santa Clara thick films. Autoscope results are given for two groups of slides: (1) in the main text, results are given for all slides, regardless of Autoscope WBC counts; (2) results are provided for slides where Autoscope imaged at least 0.1 µL of blood (i.e. WBC count ≥ 600), assuming 6000 WBCs/µL in Additional file 2. Manual microscopy results from Morona are reported for all slides, i.e. (1) above.

### WBC counts by clinic

**Fig. 3:**
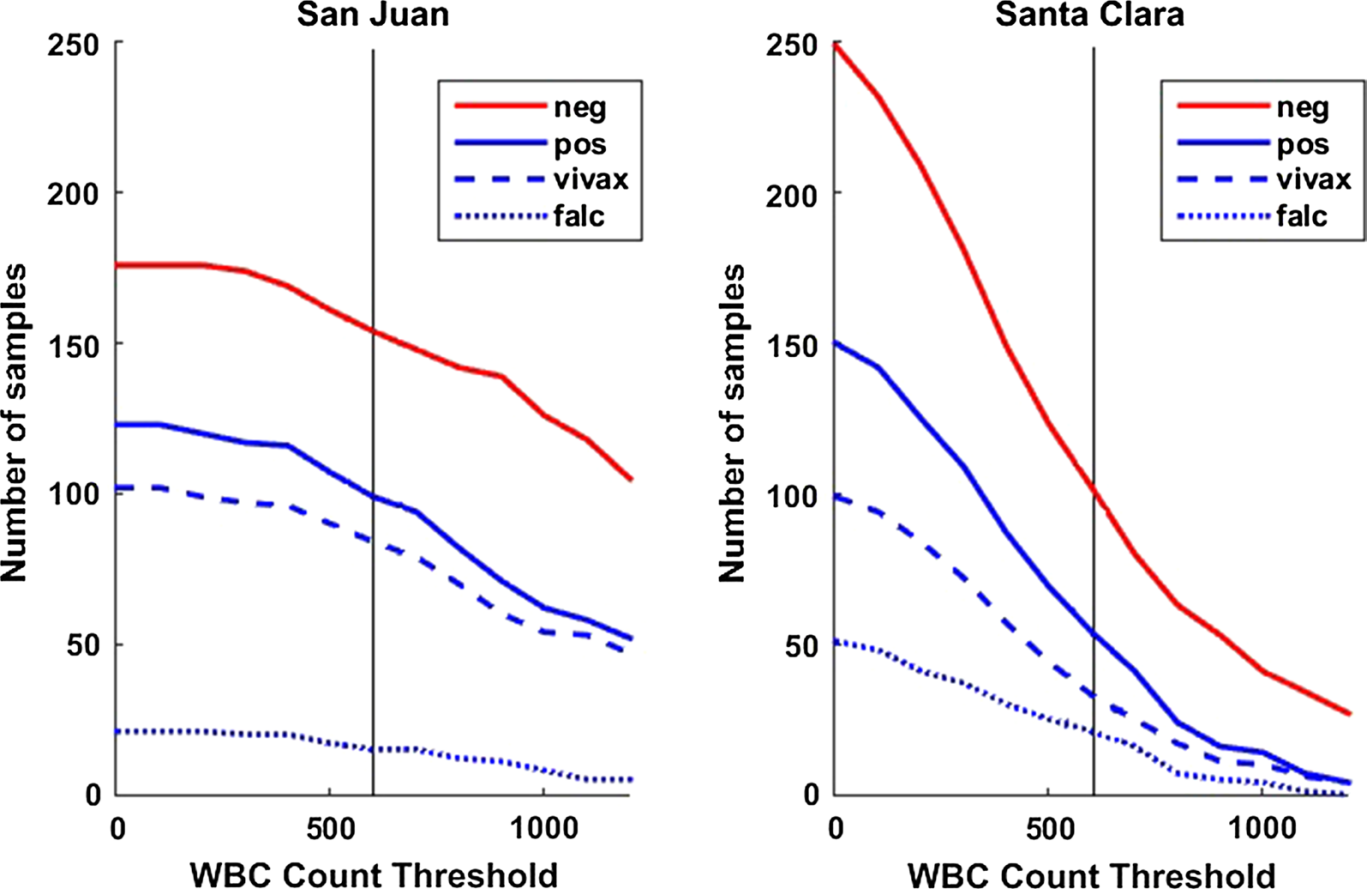
Number of slides with counts above minimum WBC count thresholds, sorted by clinic

Because Autoscope images a fixed area of the thick film (324 FoVs, approximately 2.5 mm^2^), the film thickness (volume of blood/mm^2^) determines the total volume imaged. This is approximated here by the white blood cell count. San Juan slides had WBC counts consistent with Autoscope’s design assumptions (i.e. WBC count ≥ 600 in 324 FoVs), while Santa Clara slides often had WBC counts lower than this (Fig. 3). Only 39% of Santa Clara slides (156/400) contained at least 600 WBCs in the blood imaged, compared to 84% of San Juan slides (253/300). Macro images of slides from the two clinics are shown in Additional file 3.

### Autoscope versus microscopy, using PCR as reference

**Fig. 4:**
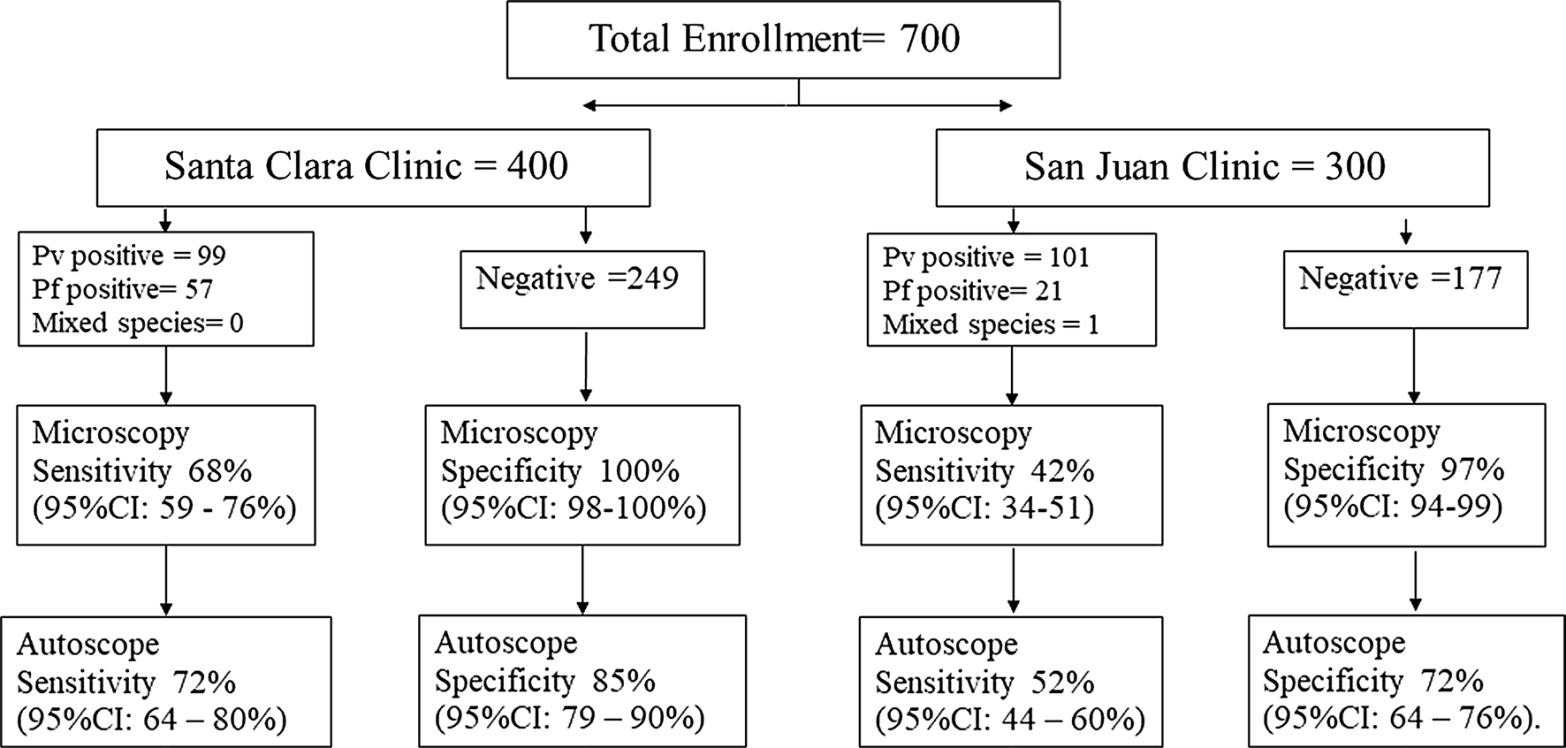
Flow chart of samples per clinic and results of microscopy vs. Autoscope using PCR as a reference

**Fig. 5:**
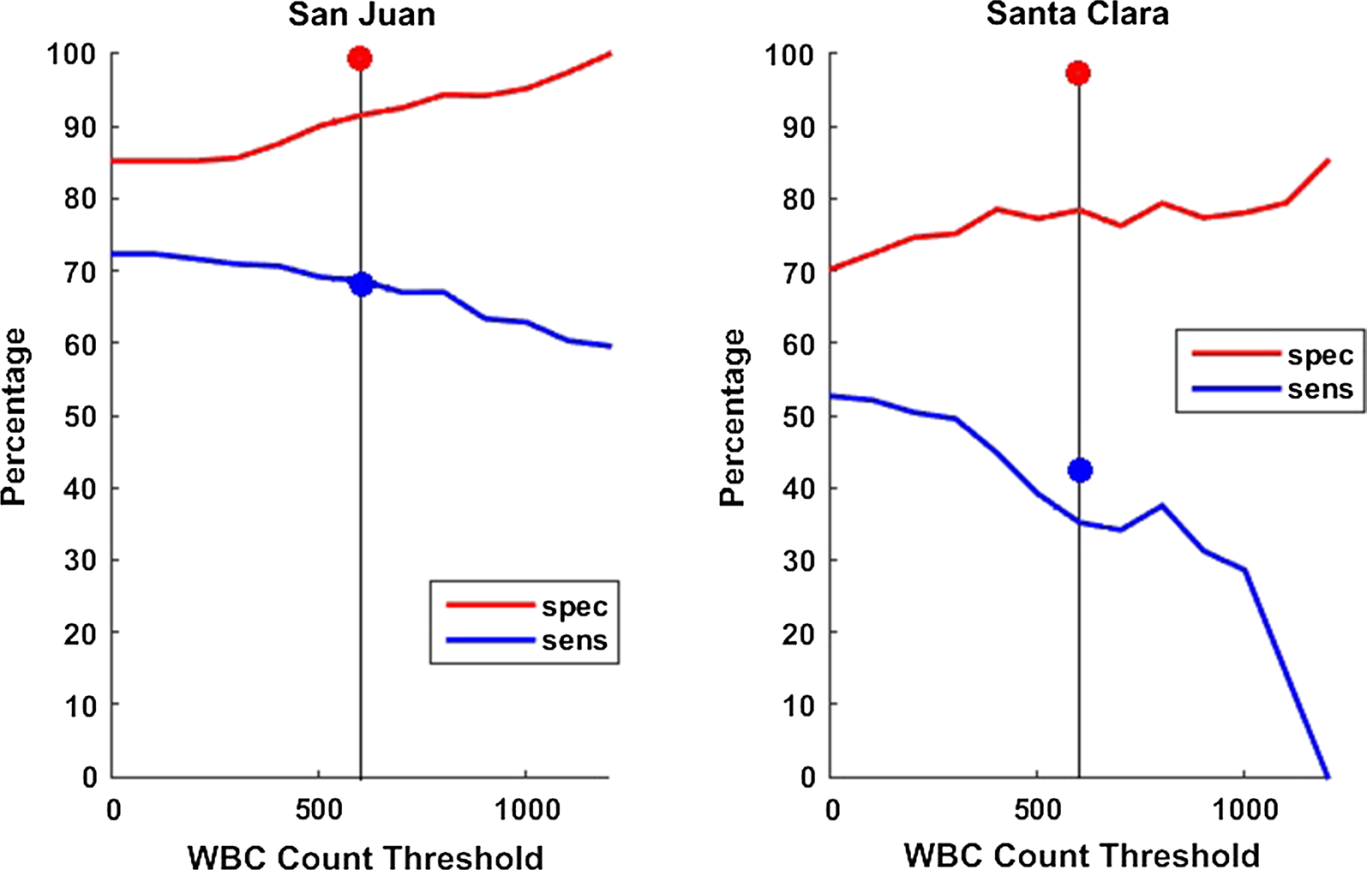
Autoscope sensitivity and specificity for all species, using PCR reference, vs. WBC count threshold

All samples were analysed by Autoscope and microscopy (Fig. 4). Samples were batched and PCR was performed at the end of the trial. Diagnostic performance of both microscopy (Table 1) and Autoscope (Table 2) differed according to clinic of origin. Microscopy and Autoscope performed better on San Juan slides. Poorer diagnostic performance by Autoscope on Santa Clara slides, relative to San Juan slides, is also evident even when samples with < 600 WBC are excluded (Fig. 5). Limits of Detection (LoD) were similar for microscopy and Autoscope on San Juan slides, ~ 100 p/uL (this estimate is limited because there were no San Juan slides with manually-counted parasitaemia < 100 p/µL). On Santa Clara slides, Autoscope LoD was ~ 200, while microscopy LoD was ~ 50 p/µL (a few Santa Clara slides had < 100 p/µL, accounting for the difference from LoD on San Juan slides). In general, the Autoscope and microscopists missed the same low-parasitaemia slides that were flagged as positive by PCR. LoD applies to thick films for both microscopy and Autoscope, since microscopy necessarily uses the thick film is used to diagnose low-parasitaemia samples.

**Table 1:** Microscopy diagnostic performance vs. PCR

	n slides (pos, neg)	Diagnostic performance % (95% CI)				
		Sensitivity	Specificity	PPV	NPV	Accuracy
San Juan						
ll species	300 (123, 177)	68 (59–76)	100 (98–100)	100 (96–100)	82 (76–87)	87 (83–91)
* P. vivax*	300 (102, 198)	70 (60–78)	100 (98–100)	100 (95–100)	86 (81–91)	90 (86–93)
* P. falciparum*	300 (21, 279)	62 (38–82)	100 (99–100)	100 (75–100)	97 (95–99)	97 (95–99)
Santa Clara						
All species^a^	400 (151, 249)	42 (34–51)	97 (94–99)	90 (81–96)	74 (68–78)	77 (72–81)
* P. vivax*	400 (100, 300)	46 (36–56)	98 (96–99)	88 (77–96)	84 (80–88)	85 (81–88)
* P. falciparum*	400 (52, 348)	31 (19–45)	100 (99–100)	100 (79–100)	91 (87–93)	91 (88–94)

Results for microscopy, separated by clinic. Sensitivity was stronger on San Juan slides. Specificity was strong on slides from both clinics

^a^One mixed species sample was detected

**Table 2:** Autoscope diagnostic performance vs. PCR

	n slides (pos, neg)	Diagnostic performance % (95% CI)				
		Sensitivity	Specificity	PPV	NPV	Accuracy
San Juan						
All species	300 (123, 177)	72 (64–80)	85 (79–90)	77 (68–84)	82 (75–87)	80 (75–84)
* P. vivax*	300 (102, 198)	72 (62–80)	83 (77–88)	69 (59–77)	85 (79–90)	79 (74–83)
* P. falciparum*	300 (21, 279)	33 (15–57)	99 (97–100)	78 (40–97)	95 (92–97)	95 (91–97)
Santa Clara						
All species^a^	400 (151, 249)	52 (44–60)	70 (64–76)	52 (43–59)	71 (65–76)	64 (58–68)
* P. vivax*	400 (100, 300)	60 (50–70)	71 (66–76)	41 (33–49)	84 (79–89)	69 (64–73)
* P. falciparum*	400 (52, 348)	4 (0–13)	99 (98–100)	50 (7–93)	87 (84–90)	87 (83–90)

Results for autoscope on all slides, separated by clinic

^a^Only one mixed species sample was detected

Comparison between Autoscope and microscopy given adequate blood volume can be seen in the results for San Juan, Additional file 3: Figure S3a. When sensitivities are similar (~ 68%), microscopy specificity was higher (98% vs 90%). Autoscope sensitivity at higher specificity (i.e. at a different point on the sens/spec tuning curve) can be inferred from values on slides with > 1000 WBCs: At this point, Autoscope has similar specificity to microscopy (98%), but lower sensitivity (60%). So trained microscopy had a performance edge.

### Autoscope quantitation concordance with expert microscopy

Similarity of parasite quantitation determined by Autoscope *versus* Morona expert microscopy varied according to the clinic of origin. To evaluate the degree of similarity, Pearson’s correlation coefficient was calculated on the log-transformed parasitaemia values of true positive slides, using PCR as the reference standard. Autoscope and microscopy parasite densities were more strongly correlated on San Juan slides, *r* = 0.86 (95% CI 0.79–0.91), compared to Santa Clara slides, *r* = 0.59 (95% CI 0.37–0.75) (Fig. 5). On the Santa Clara slides, Autoscope underestimated parasite density relative to microscopy on most slides (Fig. 5). If microscopy quantitation was done on thin films, this would naturally lead to relative underestimation by Autoscope, since thick films having lower parasitaemias [33].

### Inter-device quantitation concordance

**Fig. 6:**
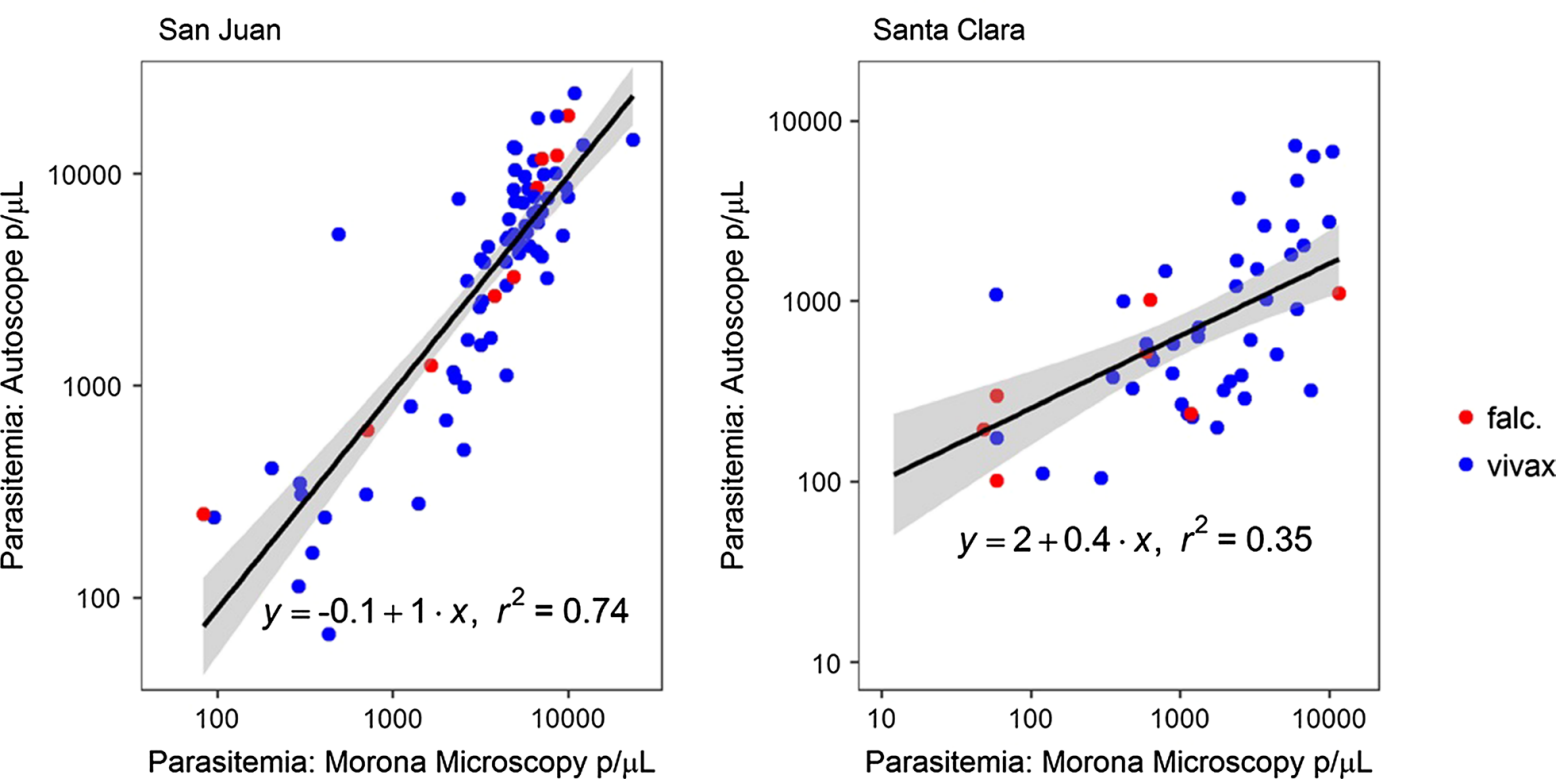
Linear regression of log-transformed parasitaemia quantitated by Autoscope vs. microscopy on true positive slides, sorted by clinic

The concordance of the Autoscope at Morona and the Autoscopes at the two clinics depended on clinic and on the number of WBCs imaged. For San Juan slides, 48 of 300 slides (16%) were discordant. For Santa Clara slides, 168 of 400 (42%) were discordant. On concordant slides, Pearson’s correlation coefficient was computed to assess the correlation between log-transformed parasitaemia values. Restricted to concordant slides, the Autoscopes at San Juan and at Morona correlated with *r *=0.90 (95% CI 0.85–0.93) on all slides, and 0.95 (95% CI 0.92–0.97) on slides with > 600 WBCS. Autoscopes at Santa Clara correlated with *r *= 0.41 (95% CI 0.26–0.55) on all slides, and *r* = 0.71 (95% CI 0.37–0.88) on slides with > 600 WBCs (Fig. 6).

### External quality control

EQC was performed to provide a subjective scale of readability. In December 2016, SMRU reviewed 70 paired field slides representing 10% of the overall study slides, selected randomly, following WHO guidelines for EQC.

Of the 70 slides, 59 slides (84.3%) were considered adequate for evaluation. Three slides (4.3%) were invalid because of stain deterioration over time (precipitate). Readable slides were cross-checked. Of the 59 readable slides, 3 slides (5.1%) diagnosed as ‘negative’ by microscopy were *P*. *falciparum* positive. Discarding the three invalid slides, slide quality issues identified included 16 slides (22.9%) which had thick films with flaws, mostly insufficient blood, and 6 slides (8.6%) that were poorly stained.

The proportion of slides with inadequate blood volume varied by clinic. Excluding invalid slides, 4 of 33 San Juan slides (12%) had inadequate volume. Of Santa Clara slides, 13 out of 34 (38%) had inadequate volume. These proportions correspond to the proportions of slides for which Autoscope counted less than 600 WBCs (San Juan) and less than 400 WBCs (Santa Clara), further confirming lower blood density on Santa Clara slides.

## Discussion

This study examined the diagnostic performance of a prototype device that uses an image recognition algorithm to identify and classify malaria parasites on routinely-prepared Giemsa-stained blood films. Results of the study reflect both the potential for artificial intelligence to perform tasks currently conducted by highly-trained experts, and the challenges of replicating the broad depth and adaptiveness of human thought processes. The prototype device was not able to compensate as well as expert microscopists on blood films with inadequate blood volume. On slides with adequate blood volume, the prototype device demonstrated that machine learning-based algorithms have potential to achieve equivalence to expert human microscopists and thus offer a viable approach to mitigating the persistent problem of poor reliability of routine malaria microscopy.

A key finding of this study was that it evaluated diagnostic performance of a prototype machine-learning device under field conditions that represent a realistic use-case for the technology. Blood films prepared at the clinics demonstrated variability, in slide preparation quality and blood volume, likely to be routinely observed were the prototype device to be used at peripheral health clinics as intended. Slides were prepared in a routine manner intended to optimize human performance, and scanned at a magnification intended for human-based identification.

Because only two clinics were included in the study, it is impossible to generalize broadly from the differences observed between the two. Diagnostic performance of the prototype device was considerably poorer, well below that of expert microscopists, on slides from one of the two clinics. It is not possible from this study to know how prevalent such conditions are, and how representative the two clinics in the study are of clinics that would use the device overall.

In addition, the algorithm in this study was trained on high-quality blood films, and it is not possible to know from this study what the diagnostic performance would have been had the algorithm been trained on films of variable quality. The high performance at San Juan suggests that the algorithm had been sufficiently trained in terms of number of correctly annotated objects parasites to correctly classify new objects, given blood films with presentation similar to that of training films. The algorithm used in this trial was trained on approximately 150 high-quality thick films with 75,000 parasites, obtained from several sites in endemic and non-endemic countries. Poorer performance at Santa Clara is likely due in part to the device not having been trained on slides with low blood volume. Autoscope thresholds were calibrated assuming approximately 0.1 µL of blood scanned; less blood scanned will lead to a larger standard error for discovered objects per WBC parasites and distractors. Low blood volume slides violate Autoscope’s design assumptions, resulting in lower specificity. Error generated by low blood volume slides could be mitigated by altering the scanning protocol to ensure sufficient blood is imaged, for example by basing scanning volume on the WBC count as a human technician is trained to do.

The differential performance of microscopists and the AI-based algorithm between the clinics is important for understanding the potential of such algorithms in diagnostic practice. An algorithm is limited by the images on which it has been trained. A microscopist takes into account a wide set of contextual conditions. Knowing the particulars of the clinic and technicians who prepared a film, noting sometimes subtle changes in overall slide color, and the ability to select certain areas of a thick film for greater scrutiny, could be included in a software algorithm. However, in practice the complexity and the often localized nature of the knowledge required make this impractical.

The Autoscope hardware performed well. Failures on Santa Clara slides were due to the software, in particular the algorithm’s lack of adaptability to irregular blood films prepared in the Santa Clara clinic. However, the Peruvian malaria control program maintains strong quality protocols. Thus the presence of difficult (for the algorithm) blood films even within the top-notch Peruvian system indicates that the issue of slide variability will occur throughout the world.

The primary use case for the Autoscope is as a research tool. While the algorithm is designed to standardize microscopy for research, the results suggest that with further improvement it may be possible to meet sufficient accuracy for case management using these methods. Given the relatively early version of the algorithm used in this study, the results hold considerable promise for replacing or enhancing certain aspects of human-based malaria microscopy with software, without changing well-established and low-cost methods for blood film preparation.

A high-performing algorithm could serve to standardize film interpretation across geography and across time, of particular relevance to clinical studies of drugs, vaccines and other diagnostics. It could also serve as a cross-checking on microscopist performance; machines do not tire, and cross-checking of slides, while recommended in any Quality Assurance (QA) programme, is rarely performed adequately [10]. In non-endemic areas, such as diagnosis of returned travelers, the device could supplement diagnosis provided by technicians who rarely see real parasites.

If the challenges of variable slide quality can be overcome, the prototype device described herein may serve as a primary diagnostic tool in areas where microscopy is still the basis for case management. Recognition and flagging of poor quality films can partially address this problem. As with other machine-learning algorithms, exposure to a larger number of well-annotated images is a proven way to improve performance, and should be considered in future research.

## Conclusion

Autoscope’s diagnostic performance was on par with routine microscopy when slides had adequate blood volume to meet its design assumptions, as represented by results from the San Juan clinic. Autoscope’s diagnostic performance was poorer than routine microscopy on slides from the Santa Clara clinic, which generated slides with lower blood volumes. Results of the study reflect both the potential for artificial intelligence to perform tasks currently conducted by highly-trained experts, and the challenges of replicating the adaptiveness of human thought processes.

## Additional files

**Additional file 1.** Supplemental information on the autoscope device.

**Additional file 2.** Autoscope diagnostic performance vs. PCR for slides with > 600 WBC (i.e., at Autoscope’s design specifications), separated by clinic.

**Additional file 3.** Typical blood films from San Juan (left) and Santa Clara (right), showing different blood densities. Santa Clara slides had much lower blood volume (fewer WBCs) per unit area.

## Abbreviations

AI: artificial intelligence
CNN: convolutional neural networks
EQC: external quality control
FOV: fields of view
NPV: negative predictive value
PCR: polymerase chain reaction
PPV: positive predictive value
QA: quality assurance
RDT: rapid diagnostic test
SMRU: Shoklo Malaria Research Unit
TES: WHO Therapeutic Efficacy Survey
UPCH: Universidad Peruana Cayetano Heredia
WBC: white blood cell
WHO: World Health Organisation
