# Supplementary material for: Automated microscopy for routine malaria diagnosis: a field comparison on Giemsa-stained blood films in Peru
Source: Malar J. 2018 Sep 25;17:339. doi: 10.1186/s12936-018-2493-0 (PMC6157053; doi:10.1186/s12936-018-2493-0)
Supplement: Supplementary file 1 — Additional file 1. Supplemental information on the autoscope device. [file 12936_2018_2493_MOESM1_ESM.docx]

# Hardware

The Autoscope scans roughly 0.1 µL of blood (given standard thick film prep), corresponding to about 300 fields-of-view (FOV) given the optics and camera. The Autoscope uses a 100× 1.25 NA oil immersion microscope, giving similar optical resolution to microscopy (~0.2 microns). This results in a depth of field of approximately 0.6 µm. Since parasites are often located at different depths, nine image slices differing by 0.3 µm in height are taken for each FOV, to ensure an in-focus image of every parasite is captured. A linear piezo accompanied by an optical (linear) encoder provides rapid motion along the z-axis with a resolution of ± 0.1 µm. Focus performance is determined using a Brenner gradient algorithm across the FOV, resulting in a focus score for each z-stack level. A white LED (1.0 watt) combined with a diffuser results in an exposure time of 8-20 ms. The images have 11.36 pixels/micron resolution, exceeding the required minimal sampling rate given the optical resolution

# Annotations

Image sets were manually annotated using software with graphical user interfaces (GUIs). Each parasite was marked with *x, y* and *z* coordinates, and given a “type” label (eg ring, late-stage, etc). Objects that were not clearly either parasites or distractors were labeled “doubtful”, and withheld from training sets. Annotations of parasites were multiply vetted by multiple on‑site and off‑site experts. Negative samples were assumed to be parasite-free. Annotations, and sample meta-data, were stored in an SQL database. Manual annotation recorded only the *x‑y* location of each parasite; best focus *z*-stack level was computed by the algorithm. Algorithm training and validation sets were organized at the sample level. For a given sample, parasites were drawn from the database, while distractors were defined as any objects detected by the algorithm which were not parasites. More details are given in [19].

# Algorithms

The Autoscope algorithm processes blood film microscopy images through five modules sequentially: image preprocessing, object detection, feature extraction & classification, arbitration, and disposition. A brief description of each of these modules follows; a full exposition of the Autoscope system may be found in [19].

The preprocessing module white balances images before further processing to eliminate some of the color differences resulting from variability in slide preparation and image acquisition. Autoscope bases and applies the white balance transform on all of the fields-of-view collectively rather than each individually, which increases system stability.

The detection module generates object proposals—objects that roughly look like parasites. The colors of malaria parasite nuclei are similar to those of WBC nuclei, both being composed of DNA and staining purple under Giemsa stain. In a first pass sweep through all the fields of view, the detection module collects pixel-level color statistics of both WBC nuclei and background. To improve object proposal performance, non-linear functions are applied to the red, green, and blue (RGB) color channels. Subsequently, the algorithm computes the optimal non-linear color projection based on this collection of labeled pixels. Dynamic, local thresholding is applied to the resulting (adaptive) grayscale image to produce the collection of object proposals. An additional step of weeding out obvious non-parasites based on low-cost attributes further improves object proposal performance.

The feature extraction and classification module is based on convolutional neural networks (CNN); specifically, we use a VGG-style architecture [SIe]. The full VGG network with 16 layers and 138M weights would lead to severe overfitting, so we use a streamlined 9-layer VGG architecture with 95K weights. The CNN automatically determines features that distinguish between the target classes on which it was trained. Two separate CNN models are employed. The first is trained to recognise immature trophozoites (referred to as ring-stage parasites), while the second is trained to recognise mature trophozoites, schizonts, and gametocytes (collectively referred to as late-stage parasites). Both are trained to distinguish between their target class, *i.e.* ring-stage or late-stage parasites, and distractors (non-parasite objects that look similar to parasites and are the by-product of the object detection module). The network weights are trained on thumbnail images of distractors and annotated parasites. The thumbnails consist of the white-balanced RGB image clips, as well as a fourth image channel that is based on the adaptive grayscale image. The number of thumbnails is augmented using the following transformations: image flips, $x, y$ translations, and color channel perturbations using gamma transforms.

The two CNN models may occasionally detect the same object. These conflicts are resolved in the arbitration module, which fixes the classification of the object based on a comparison of the output scores of the two CNN models.

The disposition module translates object-level results to a patient-level diagnosis and quantitation. The diagnosis decision is based on the number of suspected high-scoring object that exceed a count threshold. The quantitation algorithm subtracts the background false positive rate from the suspected number of parasite objects and divides by the estimated sensitivity. The parameters of the diagnosis and quantitation algorithms are determined from a validation set whose parasites have been annotated and vetted by multiple experts.

SI_a. Y. LeCun, B. Boser, J. S. Denker, D. Henderson, R. E. Howard, W. Hubbard, and L. D. Jackel. Backpropagation applied to handwritten zip code recognition. Neural Computation 4:541-551, 1989.

SI_b. A. Krizhevsky. I. Sutskever, G.E. Hinton. ImageNet Classification with Deep Convolutional Neural Networks. Advances in Neural Information Processing Systems, 25:1097-1105, 2012.

SI_c. D.C. Ciresan, A. Giusti, L.M. Gambardella, J. Schmidhuber. Mitosis Detection in Breast Cancer Histology Images with Deep Neural Networks. In International Conference on Medical Image Computing and Computer-assisted Intervention, pp. 411-418. Springer Berlin Heidelberg, 2013.

SI_d. K. He X. Zhang, S. Ren, J. Sun. Deep Residual Learning for Image Recognition. In IEEE Conference on Computer Vision and Pattern Recognition, 2016.

SI_e. K. Simonyan, A. Zisserman. Very Deep Convolutional Networks for Large-Scale Image Recognition. arXiv:1409.1556 [cs.CV], 2015.
