# Supplementary material for: Automated microscopy for routine malaria diagnosis: a field comparison on Giemsa-stained blood films in Peru
Source: Malar J. 2018 Sep 25;17:339. doi: 10.1186/s12936-018-2493-0 (PMC6157053; doi:10.1186/s12936-018-2493-0)
Supplement: Supplementary file 2 — Additional file 2. Autoscope diagnostic performance vs. PCR for slides with > 600 WBC (i.e., at Autoscope’s design specifications), separated by clinic. [file 12936_2018_2493_MOESM2_ESM.docx]

**Additional File 2: Autoscope diagnostic performance vs. PCR for slides with >600 WBC (i.e., at Autoscope’s design specifications), separated by clinic.** Autoscope sensitivity and specificity was higher on San Juan slides.

|  |  | **Diagnostic Performance % (95%CI)** | | | | |
| --- | --- | --- | --- | --- | --- | --- |
| **San Juan** | **n slides**  **(pos, neg)** | **Sensitivity** | **Specificity** | **PPV** | **NPV** | **Accuracy** |
| all species | 253 (99, 154) | 69 (59 to 78) | 92 (86 to 95) | 84 (74 to 91) | 82 (75 to 87) | 83 (77 to 87) |
| *P. vivax* | 253 (84, 169) | 68 (57 to 78) | 90 (84 to 94) | 77 (66 to 86) | 85 (79 to 90) | 83 (77 to 87) |
| *P. falciparum* | 253 (15, 238) | 33 (12 to 62) | 99 (97 to 100) | 71 (29 to 96) | 96 (93 to 98) | 95 (92 to 98) |
|  |  |  |  |  |  |  |
| **Santa Clara** | **n slides**  **(pos, neg)** | **Sensitivity** | **Specificity** | **PPV** | **NPV** | **Accuracy** |
| all species* | 156 (54, 102) | 35 (23 to 49) | 78 (69 to 86) | 46 (31 to 63) | 70 (60 to 78) | 63 (55 to 71) |
| *P. vivax* | 156 (33, 123) | 48 (31 to 66) | 79 (71 to 86) | 38 (24 to 54) | 85 (77 to 91) | 72 (65 to 79) |
| *P. falciparum* | 156 (21, 135) | 0 (0 to 16) | 100 (97 to0) | NA | 87 (80 to 91) | 87 (80 to 91) |

*One mixed species sample was detected.
