# Supplementary figures and images for: Automated microscopy for routine malaria diagnosis: a field comparison on Giemsa-stained blood films in Peru
Source: Malar J. 2018 Sep 25;17:339. doi: 10.1186/s12936-018-2493-0 (PMC6157053; doi:10.1186/s12936-018-2493-0)

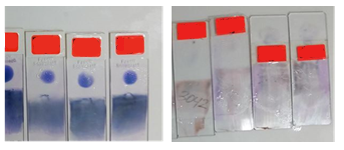

Supplement: Supplementary file 3 — Additional file 3. Typical blood films from San Juan (left) and Santa Clara (right), showing different blood densities. Santa Clara slides had much lower blood volume (fewer WBCs) per unit area. [file 12936_2018_2493_MOESM3_ESM.png]
